# Correction: The genetic identity of neighboring plants in intraspecific mixtures modulates disease susceptibility of both wheat and rice

**DOI:** 10.1371/journal.pbio.3002382

**Published:** 2023-11-08

**Authors:** Rémi Pélissier, Elsa Ballini, Coline Temple, Aurélie Ducasse, Michel Colombo, Julien Frouin, Xiaoping Qin, Huichuan Huang, Jacques David, Florian Fort, Hélène Fréville, Cyrille Violle, Jean-Benoit Morel

The ninth author’s name is incorrect. The correct name is: Jacques David.

The tenth author’s name is incorrect. The correct name is: Florian Fort.

The eleventh author’s name is incorrect. The correct name is: Hélène Fréville.

The twelfth author’s name is incorrect. The correct name is: Cyrille Violle.
