# Supplementary figures and images for: The genetic identity of neighboring plants in intraspecific mixtures modulates disease susceptibility of both wheat and rice
Source: PLoS Biol. 2023 Sep 12;21(9):e3002287. doi: 10.1371/journal.pbio.3002287 (PMC10497140; doi:10.1371/journal.pbio.3002287)

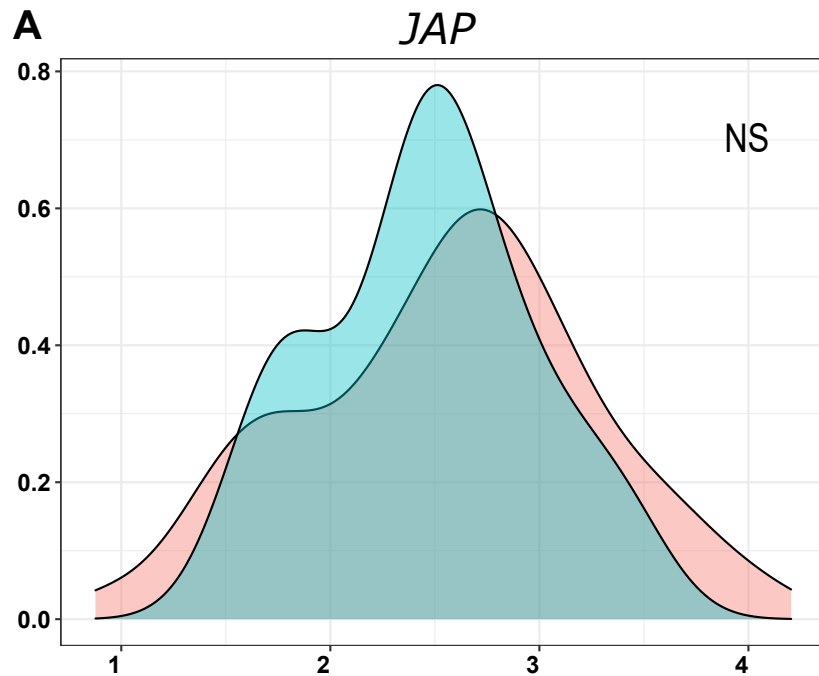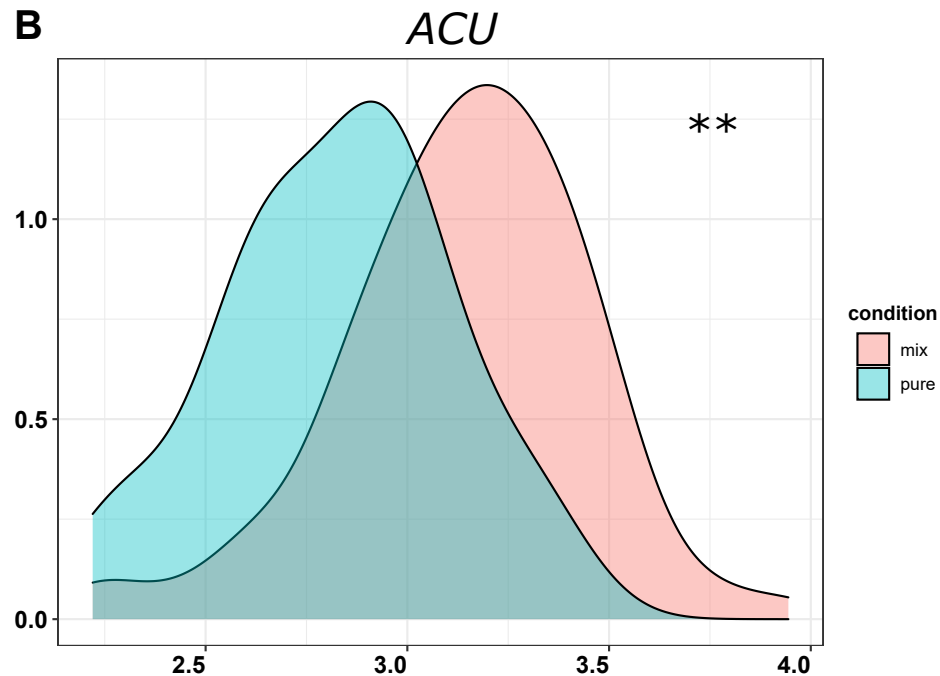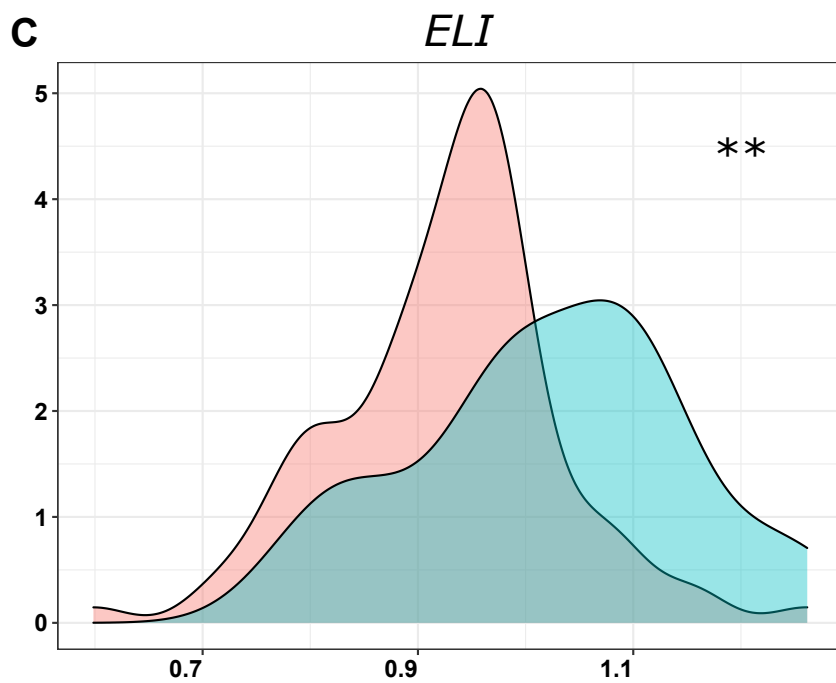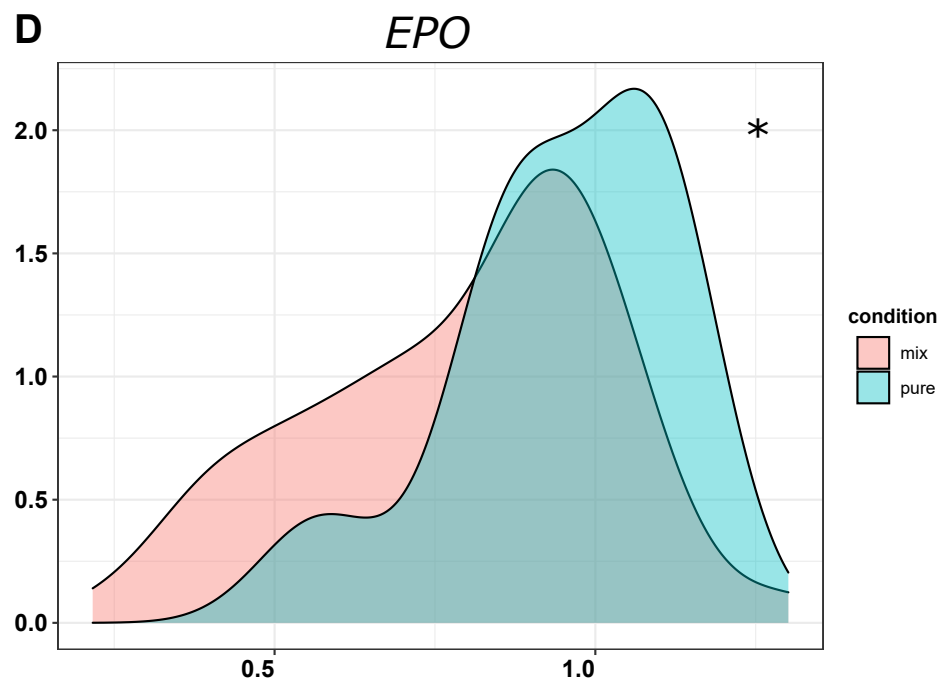Numbers of lesions/ leaf cm<sup>2</sup> (sqrt)

Supplement: S1 Fig — Disease susceptibility means (number of lesions / leaf cm2) were quantified for each focal plant in the 2 different conditions: Pure stand (pure) in blue and mixture (mix) in red. LSmeans were calculated according to Model A described in Method. LSmeans density is represented for each set of genotypes. A: Elite temperate japonica (JAPrice) (n = 132 mix and 12 pure); B: Acuce lines (ACUrice) (n = 90 mix and 10 pure); C: Elite Durum wheat (ELIwheat) (n = 90 mix and 10 pure); D: lines from pre-breeding population (EPOwheat) (n = 90 mix and 10 pure). A star symbol indicates a statistical difference detected by ANOVA performed on the model A described in Methods between the pure and the mix conditions (NS: p > 0.1; *: p < 0.05; **: p < 0.01, ***: p < 0.001). The data used can be found at https://doi.org/10.57745/RRA3HL. (PDF) [file pbio.3002287.s001.pdf]

**A***JAP*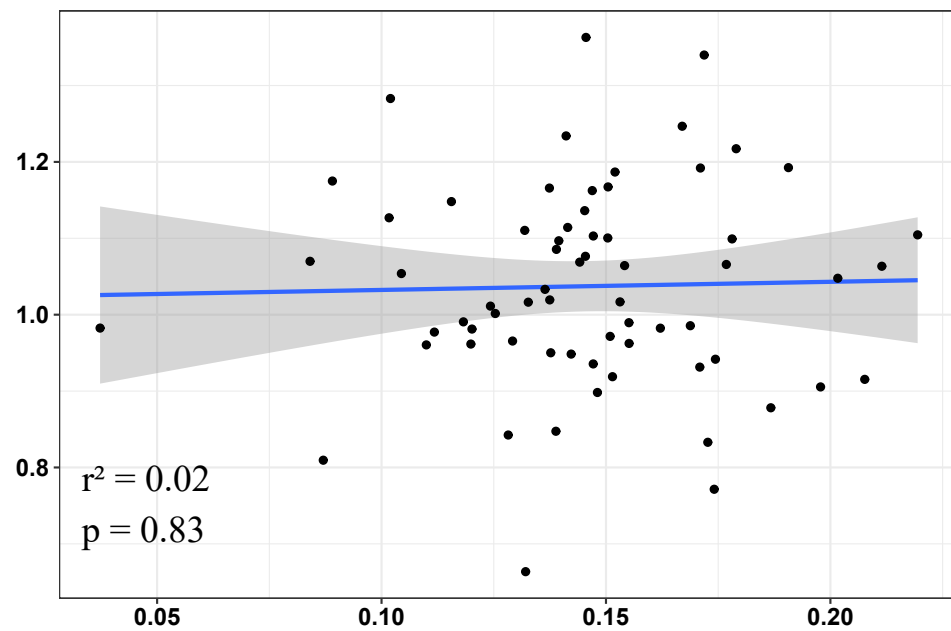**B***ACU*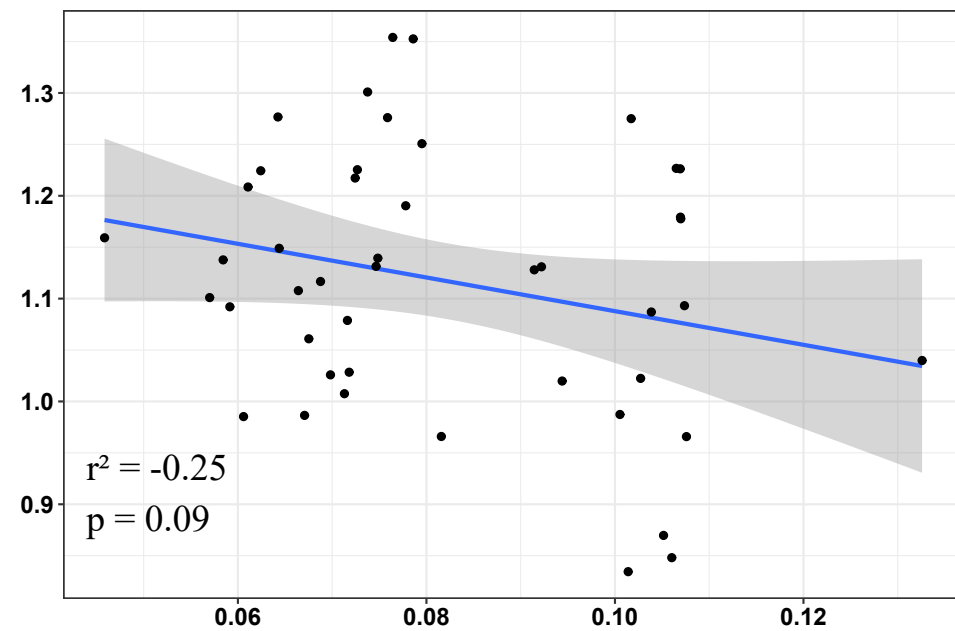**C***ELI*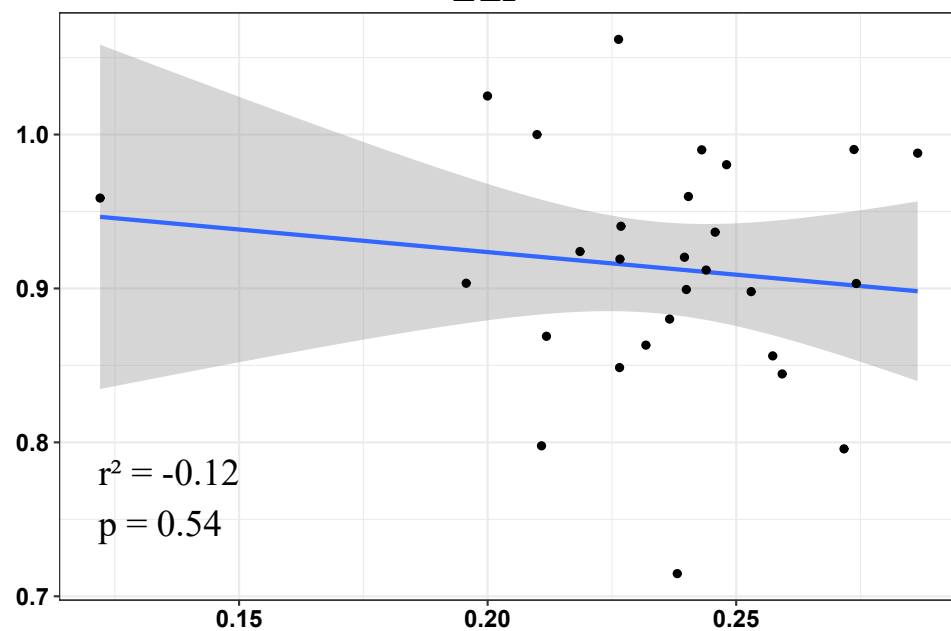**D***EPO*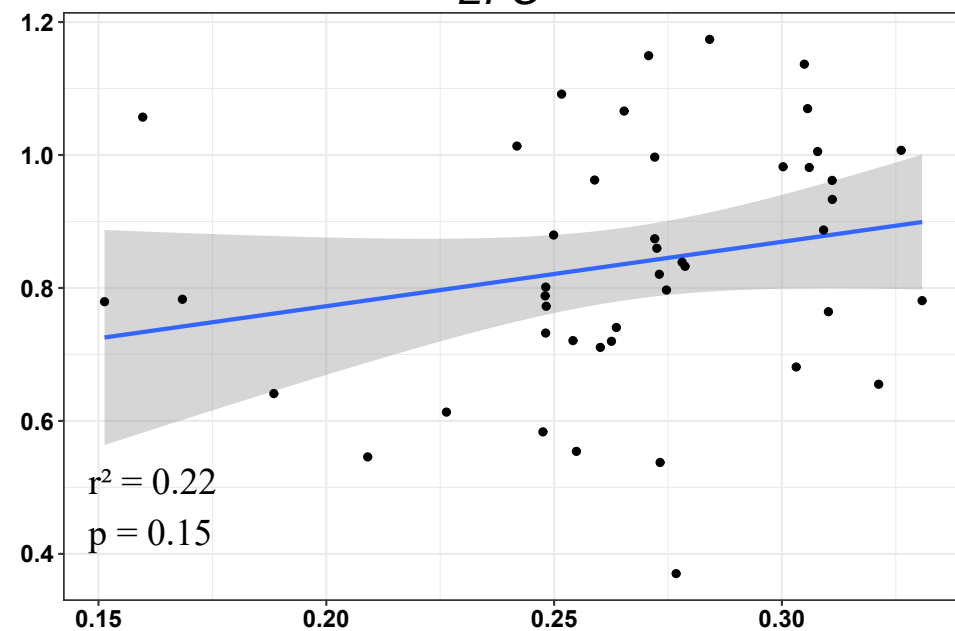

Genetic distance

Supplement: S2 Fig — Modulation of disease susceptibility in focal is the fold change of susceptibility in focal plant by a neighbor genotype compared to its value in pure stand. For each set of genotypes, Elite temperate japonica (A: JAPrice, n = 132), Acuce lines (B: ACUrice, n = 90), Elite Durum wheat (C: ELIwheat, n = 90), and Pre-breeding population (D: EPOwheat, n = 90), this modulation of disease susceptibility in focal was compared to the susceptibility value of the corresponding neighbor. Pearson correlation were calculated and R2 and p.value are shown. The data used can be found at https://doi.org/10.57745/RRA3HL. (PDF) [file pbio.3002287.s002.pdf]

**A***JAP*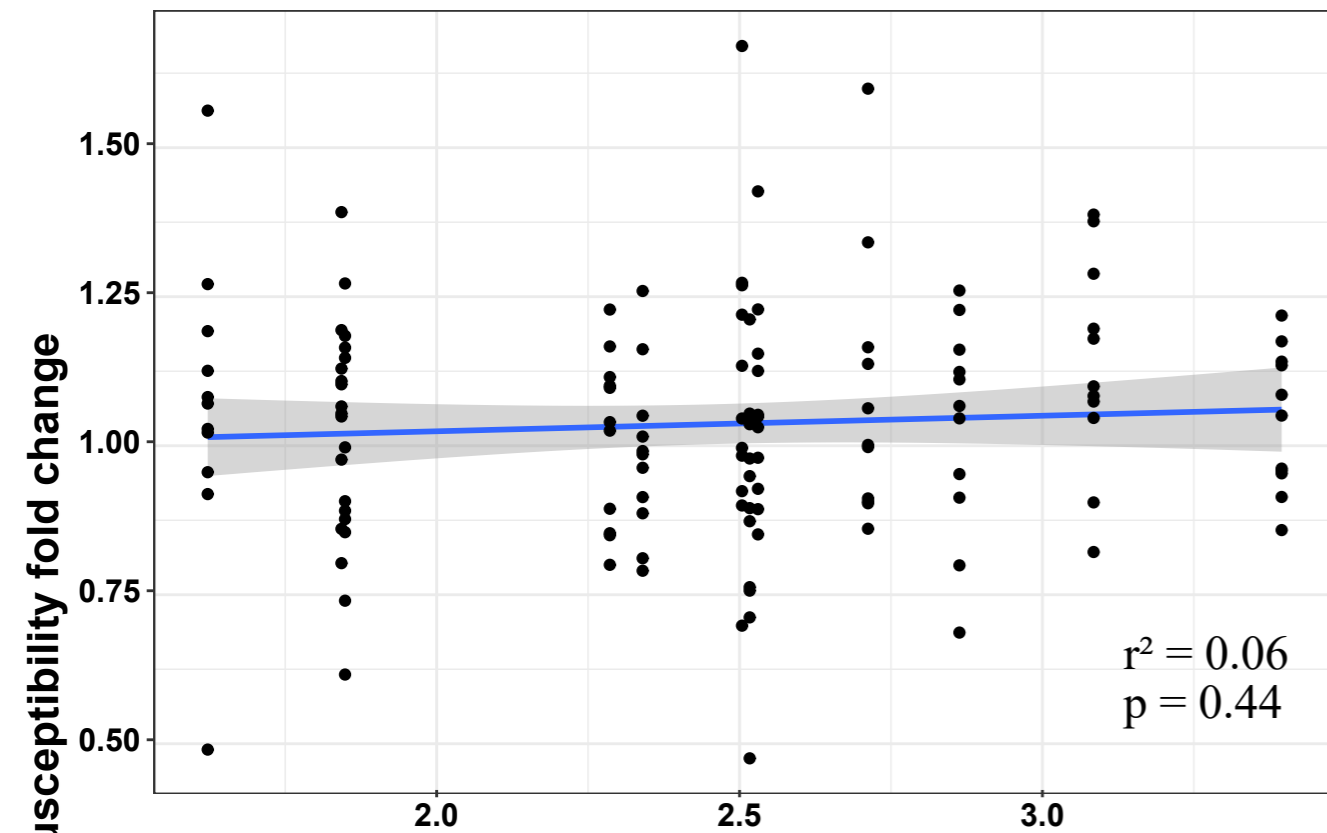**B***ACU*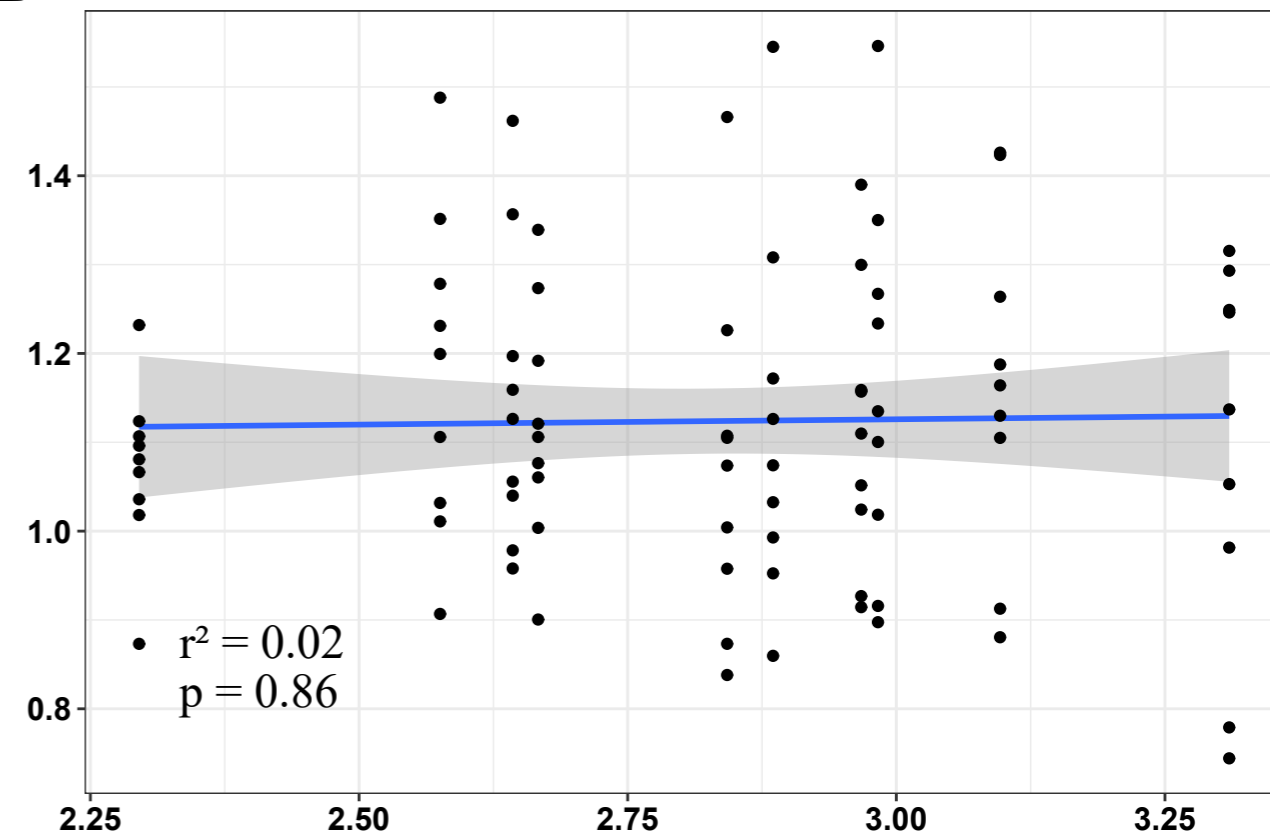**C***ELI*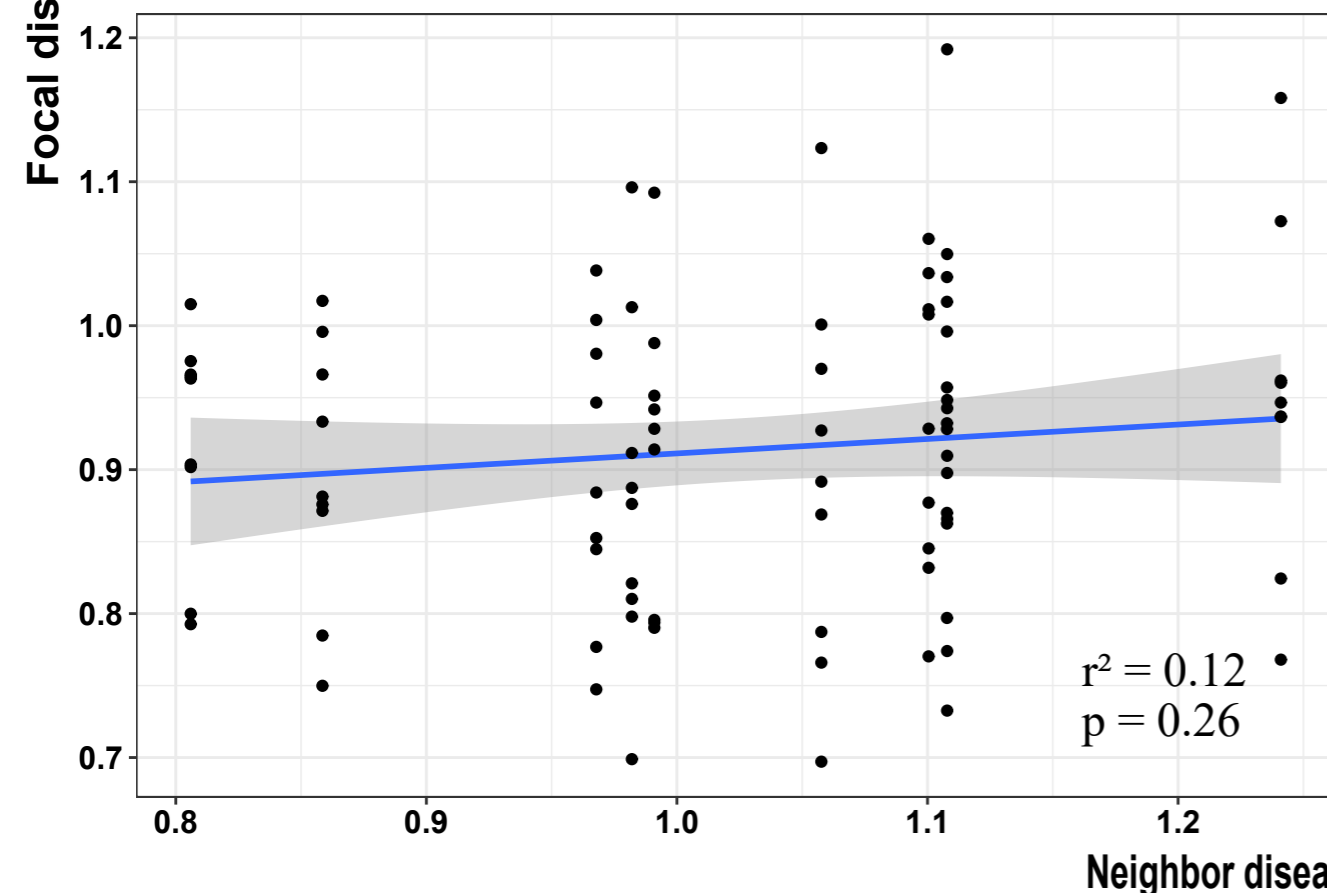**D***EPO*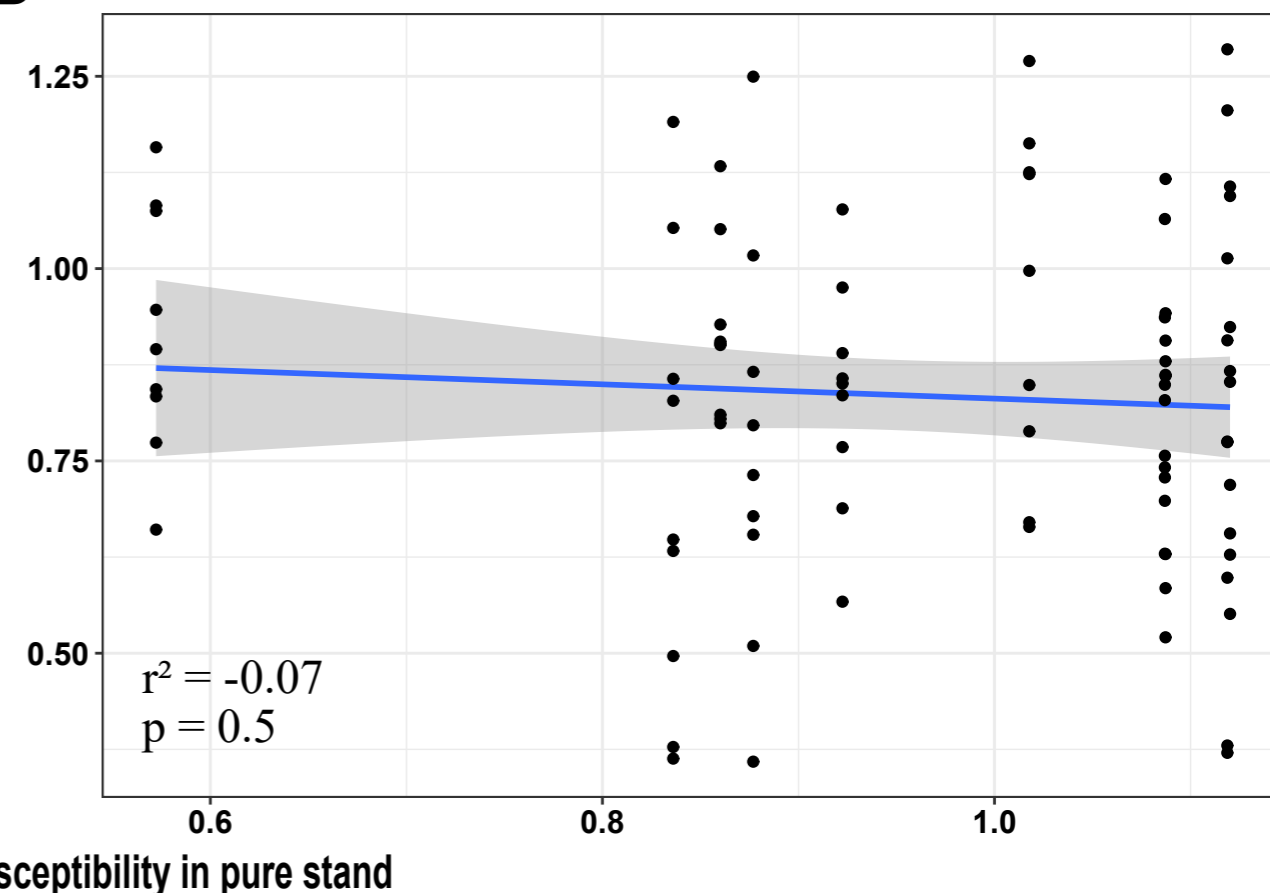

Supplement: S3 Fig — RST values represent the ratio between the average susceptibility of the 2 genotypes in mixture divided by the mean of the susceptibility of the 2 component genotypes in pure stands (see Methods). For each pair of 2 genotypes, we use a shared allele index as proxy of the genetic distance (see Methods). Relation between RST and genetic distance are shown for (A) Elite temperate japonica (JAPrice) (n = 66), (B) Acuce lines (ACUrice) (n = 45), (C) Elite Durum wheat (ELIwheat) (n = 45), and (D) Pre-breeding population (EPOwheat) (n = 45). Pearson correlation were calculated and R2 and p.value are shown. The data used can be found at https://doi.org/10.57745/RRA3HL. (PDF) [file pbio.3002287.s003.pdf]
