# Supplementary material for: The genetic identity of neighboring plants in intraspecific mixtures modulates disease susceptibility of both wheat and rice
Source: PLoS Biol. 2023 Sep 12;21(9):e3002287. doi: 10.1371/journal.pbio.3002287 (PMC10497140; doi:10.1371/journal.pbio.3002287)
Supplement: Attachment — Submitted filename: Detailed answers to reviewers Pelissier et al.docx [file pbio.3002287.s005.docx]

**Pelissier et al**

**Detailed answers to reviewers**

The initial comments by the reviewers are subdivided in Points (P#). Our answers are indicated in grey and referenced as P# in the document. Consequently, the line numbers indicated by the reviewers are no longer valid but can be easily found using the P#.

**Reviewer #1: Plant genomics.**

The study investigates how plant diversity could increase mean fitness and pathogen resistance. Specifically they test so called Neighbor-Modulation of Susceptibility, where the genotype of the paired neighbor plant in the pot has an effect on the susceptibility of the focal plant in the pot.  They look at elite and unselected inbread cultivar sets of rice and wheat and compare hundreds of pairs in pots.  The study aims to look at genetic neighbor effects (direct, indirect and interaction) beyond 'classical dispersal barrier' indirect effects when mixtures are planted together in the field.  Indeed both plants in the pot should recieve equal inoculations of pathogen growing under greenhouse conditions to support this.

P1a) The genotypes are compared in a full pairwise matrix for each population and species, with mono-genotype 'controls' relatively under represented along the diagonal. The central measure if Relative Susceptibility Total index, a ratio contrasting the average susceptibility of the reciprocal pairs, to the average of the pure pots. Variation in the denominator can be high from a smaller sample and ratio statistics have large, non normal variance.

Answer to P1a) Considering the under representation of the control pots (“pure stand”): as described in Methods, our experimental design allows us to consider each genotype in binary-genotype/association as a focal and a neighbor. For pure stand pots, this implies that each genotype was screened twice. For example for rice, this corresponds to 8 individuals plants screened in each pot, in 4 pot replicates and in 3 separate experiments, totalizing 8x4x3 = 96 measures; thus the values for mono-genotype controls of average susceptibility makes the denominator a stable metrics. This was already indicated in Methods and we added a sentence to reinforce that point when describing the RST ratio:” Since pure stand controls (Sii and Sjj) were made of twice the same genotype, the Sii and Sjj values were produced with twice values than Sij and Sji”.

P1b) log difference, would be a more stable metric. Are the results subject to this transform, potentially better than the sqrt adjustment.

Answer to P1b) Relative Susceptibility Total index (ratio) was used for the purpose of representation in fig.1,as further indicated now in the Methods.

Considering the distribution of ratios, we made all our statistical analysis on the raw susceptibly data using our model A and model B, as described in Methods. This model has been elaborated with the sqrt transformation, allowing us to obtain normal distribution, in contrast with all other transformations tested; this point was already raised and solved in our previous articles (Pelissier et al, J Exp Bot 2021) and more recent (Pelissier et al, New phytol 2023) articles. Moreover, sqrt transformation makes strong corrections, thus eliminating some differences that are weakly significant. See also P20.

P2) Never the less, in table 1, only the unselected lines of rice and wheat, showed significant direct by indirect genetic interaction effects, and they were in opposite directions according to Fig1 and 2.  It is strange the ACUrice did not show direct genetic effects on susceptibility, was this significant when indirect effects were excluded?

Answer to P2) The Acuce rice genotypes all come from a unique landrace, whereas the other sets used have a broader genetic background. The low DGE observed in ACU population is thus a consequence of this situation. A sentence was added in the Result section: “These low levels of DGE may simply result from the fact that all ACU lines come from a unique landrace”.

P3) Next specific pairs were tested for neighbor effects and some lines were identified as interacting with multiple other pairs.  These should be verified in a further experiment. To go further a mapping experiment (F2, locus by line) may even be able to isolate the interacting genetic factor if this was a truly major heritable interacting factor.

Answer to P3) This work was under submission at the time of the submission of the current manuscript. We used a GWAS analysis to identify the loci in the neighbour that determine the modification of susceptibility in focal plant Kitaake (Temperate Japonica rice). Our study demonstrates that NMS in rice focal plants is controlled by one major locus in the genome of its neighbour. This work is now published since January 2023 (<https://doi.org/10.1111/nph.18778>). A sentence was added in the discussion: “Recently, we demonstrated that NMS is amenable to genetics and identified a locus in rice neighbors that modulate resistance in its neighborhood (33).”

P4) Another follow up experiment would be to contrast pairs of elite vs unselected cultivars to test the hypothesis that selection has removed this neighborhood modulation potential. It is unexpected that genetic distance among lines did not predict NMS.

Answer to P4) We agree that our negative finding that genetic diversity did not predict NMS was unexpected. However, the experiment suggested is not ecologically relevant considering that the sets used here are not found in similar environments. For instance, it is not possible to find Acuce rice (i.e indica) and elite line of temperate japonica grown together and we anticipated this would result in artefacts. The demonstration a possible link between genetic distance and NMS will require a specific experiment and this was not the intent of this work. We figured some reviewers would ask for this analysis, and this why we provided this result as a Suppl fig. If requested we can remove this information. This comment is linked to comment P6 and P7. A sentence was added in the Methods section:” Each matrix consisted in all possible pairs within each set of genotypes. Pairs of genotypes from different sets were not tested as these sets are not ecologically/agronomically compatible” as well as in the introduction: “Since the sets of genotypes occupy different ecological/agronomical environments, we only tested mixtures within each set of genotypes and avoided possible artefacts resulting from mixing genotypes from different sets”.

I am not yet convinced "our study273 brings a proof of concept.." I would like to see alternative analysis and follow up experiments which would be a standard for PLOS Biology

We changed the sentence “our study brings a proof of concept…” by ‘our study suggests that…”

**Reviewer #2: Evolution and ecology.**

In this manuscript, the authors follow up on a previous paper (Pelissier et al 2021a) in which they showed that susceptibility to disease in plants (specifically rice blast and wheat rust) depends on whether they are cultivated in pure stands or in mixtures. In short, the neighboring presence of nonself conspecifics may increase the baseline defense level of the plant, thereby decreasing its susceptibility to disease compared to a pure stand in which all plants are genetically identical. Although opposite outcomes and alternative interpretations are possible, these results shed new light on the mechanisms enabling cultivar mixtures to decrease disease prevalence as compared to pure stands. While (Pelissier et al 2021a) considered 20 pairs of plant genotypes (10 for wheat and 10 for rice), the present manuscript considers 201 pairs of plant genotypes (90 for wheat and 111 for rice). The larger size of the experiment allows the authors to go further than

(Pelissier et al 2021a) in several respects.

They first show that in wheat, mixing has an overall negative effect on disease severity (the number of lesions per unit area), meaning that plant-plant interactions can be beneficial to growers. By contrast, mixing has an overall positive effect on disease severity in rice, which cautions against plant-plant interactions. Using linear statistical models, the authors decompose the effects of the focal plant genotype, the genotype of its neighbor, and possible interactions between neighbor and focal genotypes, on disease severity. In particular, they show that the overall effect of interactions between plant genotypes on disease severity is far from being negligible. When plant-plant interactions decrease disease severity, their effect can be comparable to that of basal immunity. Plant-plant interactions can also increase severity though. The authors show which genotypic combinations promote or challenge the effectiveness of host mixtures.

I am a theoretical plant disease epidemiologist and I cannot judge the technical correctness of the experimental methods, including the statistical ones (which seem relatively standard). I accepted to perform this review because I was interested in the topic, and I did find the manuscript interesting and pleasant to read. The authors made an original and refreshing (not to say provocative) contribution to the field of plant disease epidemiology, showing that epidemiology (i.e., interactions between infected and healthy plants) may not be that relevant to explain the effectiveness (or lack thereof) of varietal mixtures!

My comments below are intended to improve the readability and impact of the article on a wide readership (including theoreticians):

P5) L69: I would recommend recalling the results and limits of the previous study (Pelissier et al, 2021) in more detail to better show the novelty of the present one.

Answer to P5) We added a new sentence: “However, this study was limited to one focal plant and the general response to neighbors in a variety of focal plants is unknown”.

P6) L88-91: On first reading I thought you would mix modern and "traditional" varieties but in fact you only mix modern varieties with modern varieties and traditional varieties with traditional varieties. It is unclear why you didn't mix modern and traditional varieties: could you explain?

Answer to P6) This comment matches the comment P4 by reviewer 1. We added a sentence in the Introduction to explain why we did not do the suggested experiment: “Since the sets of genotypes occupy different ecological/agronomical environments, we only tested mixtures within each set of genotypes and avoided possible artefacts resulting from mixing genotypes from different sets”.

We also added a sentence in the Results section: “Each matrix consisted in all possible pairs within each set of genotypes. Pairs of genotypes from different sets were not tested as these sets are not ecologically/agronomically compatible”.

P7) L102: I found it unclear on first reading which matrices you were referring to and why 4 (see my previous comment). This is because the Methods arrive after the Results. Please explain a little bit more.

Answer to P7) A sentence was added to makes this clearer and we also indicated that pairs between sets were not tested to remain consistent with initial agronomical/ecological niches. See Pa and P6 for more details.

P8) L179-182: could you please explain why your statistical design did not allow you to test for overall responsiveness to neighbors?

Answer to P8) We added a sentence: “Because of the over-representation of the mix condition (11 or 9) compared to the pure control condition (1),…”

P9) L236: I would refer to an [apparent] cooperative behaviour since neighbor-induced reduced susceptibility may be due to intraspecific competition between plants though an elevated level of defense. I realize all this is likely explained in (Pélissier et al 2021b), but I would recommend briefly recalling the possible biological interpretations behind your statistical results. For instance, how do you interpret neighbor-induced increased susceptibility in rice? In its current version, the manuscript is a bit too focused on the statistics, leaving biological interpretations implicit.

Answer to P9) We added a sentence: “Among all the mechanisms that could explain NMS, (presented in [16]), we cannot exclude a case of intra-specific competition for resource”

Minor corrections/suggestions:

P10) L62: you might consider citing Clin et al 2021.

Answer to P10) we agree and included the reference.

P11) L79-81. This sentence is redundant with L75-77.

Answer to P11) we deleted it

P12) L83: it is unclear what resistance means in this context. I would rather write "susceptibility as measured in absence of neighbor".

Answer to P12) we agree and changed the text accordingly

P13) L119: you mean a positive (not negative) effect on susceptibility, even though the effect is negative for the grower.

Answer to P13) We changed the sentence accordingly

P14) L138: do you mean [genetic/specific] interactions? Otherwise, it sounds like a tautology.

Answer to P14) We changed the sentence accordingly

P15) L172: focal [plant] here and elsewhere

Answer to P15) We added “plant” whenever needed

P16) L228: you wrote 100% line 175?

Answer to P16) The 100% reduction was found for the EPO line 6 cultivated with the neighbor 67, but this value is sub significant (p < 0.1). We corrected value in L175 to keep consistent.

P17) L256, 261: I would suggest writing epidemiological [interactions] instead of [barriers]. In this context, the term "barrier" has a specific meaning, which is too restrictive. Also, the term [barrier] sounds as if mixtures contained epidemiological obstacles while the reason why they are effective in some cases is not that clear (as indicated in the introduction).

Answer to P17) We changed the wording as suggested

P18) L303: how many plants per pot?

Answer to P18) The answer is provided in line 306

P19) L306-309: why is plastic important? What are the other pots made of?

Answer to P19) All the pots of this papers (for wheat and for rice) are made of plastic and other material exist (clay, recycled/bio-degradable fiber…), this is why we specified.

L312: “each experiment represent” was changed to “each experiment represents”

P20) L352-353: I realize that this is likely uninteresting but you should nevertheless show how making the square-root transformation corrects for normality and homoscedasticity.

Answer to P20) We showed how square roots transformation can be used in lesion counting in our previous article (17) and this was indicated.

P21) L372: why gamma and not S as before?

Answer to P21) Indeed, this was changed

P22) Fig. 1: the values on the y-axis do not seem to correspond to numbers of observations (since they can be lower than 1).

Answer to P22) Indeed this is a mistake, the right Y axis value is Density (Nb of observations / total), a correction has been made on the figure and the legend.
