# Supplementary material for: The genetic identity of neighboring plants in intraspecific mixtures modulates disease susceptibility of both wheat and rice
Source: PLoS Biol. 2023 Sep 12;21(9):e3002287. doi: 10.1371/journal.pbio.3002287 (PMC10497140; doi:10.1371/journal.pbio.3002287)
Supplement: Attachment — Submitted filename: response to reviewers Pelissier accepted.docx [file pbio.3002287.s006.docx]

**Response to reviewers**

Editor’s recommendation

Initial title of revised version: “Genetic identity of neighbors mediates disease susceptibility to pathogens in plant mixtures”

Suggested titles by Editor:

"The genetic identity of neighboring plants in intra-specific mixtures mediates disease susceptibility of both wheat and rice"

"The genetic identity of neighboring plants in intra-specific mixtures modulates disease susceptibility of both wheat and rice"

We preferred the version with “modulates” rather than the one with “mediates” as the phenomenon under study has been coined NMS (Neighbor modulated susceptibly)

Data depository

The data (now called S1_Data) used for can be found at <https://doi.org/10.57745/RRA3HL>.

Reviewer #1: The authors have addressed my initial concerns, particularly with the publication of another validation study:
“Answer to P3) This work was under submission at the time of the submission of the current
manuscript. We used a GWAS analysis to identify the loci in the neighbour that determine the
modification of susceptibility in focal plant Kitaake (Temperate Japonica rice). Our study
demonstrates that NMS in rice focal plants is controlled by one major locus in the genome of its
neighbour. This work is now published since January 2023 (https://doi.org/10.1111/nph.18778). A
sentence was added in the discussion: "Recently, we demonstrated that NMS is amenable to
genetics and identified a locus in rice neighbors that modulate resistance in its neighborhood (33).”

Response to reviewer #1: no response needed

Reviewer #2: The authors have satisfactorily addressed all my comments but a minor one, see below.

L247: I suggested inserting "apparent" just before "cooperative". Apparent competition occurs when two species that have no direct negative interactions share a common predator; increasing the abundance of one species increases the predator abundance, which indirectly decreases the abundance of the other species. Here, two plant genotypes share a common pathogen; the presence of a distinct plant genotype might increase the defense level of the focal genotype, resulting, as a by-product, in a decrease in susceptibily to pathogen infection. Although the plant genotypes may have no direct positive interactions (they may not cooperate), they may have indirect positive interactions through a shared pathogen. To me the results do not necessarily suggest cooperative behaviour in plants; they may equally suggest pathogen-mediated apparent cooperation due to plant-plant competition (the opposite of cooperation!). Writing "apparent cooperation" would be more prudent.

Authors’ response: we fully agree with this comment and made the addition L247

Other comments/misprints:

Authors’ response: we made all requested minor changes below

L57: I would suggest replacing "Many different" with "Several"

L70: please specify "genotype" just after "plant" for clarity

L80: replace ".." with "."

L207: replace ", it" with ". It"

L271: remove "pathogen"

L373: identifY

L532: remove "for each"
